# Artificial Intelligence in Drug Design

**DOI:** 10.3390/molecules23102520

**Published:** 2018-10-02

**Authors:** Gerhard Hessler, Karl-Heinz Baringhaus

**Affiliations:** 1 R&D, Integrated Drug Discovery, Industriepark Hoechst, 65926 Frankfurt am Main, Germany; 2 R&D, Industriepark Hoechst, 65926 Frankfurt am Main, Germany; karl-heinz.baringhaus@sanofi.com

**Keywords:** artificial intelligence, deep learning, neural networks, property prediction, quantitative structure-activity relationship (QSAR), quantitative structure-property prediction (QSPR), de novo design

## Abstract

Artificial Intelligence (AI) plays a pivotal role in drug discovery. In particular artificial neural networks such as deep neural networks or recurrent networks drive this area. Numerous applications in property or activity predictions like physicochemical and ADMET properties have recently appeared and underpin the strength of this technology in quantitative structure-property relationships (QSPR) or quantitative structure-activity relationships (QSAR). Artificial intelligence in de novo design drives the generation of meaningful new biologically active molecules towards desired properties. Several examples establish the strength of artificial intelligence in this field. Combination with synthesis planning and ease of synthesis is feasible and more and more automated drug discovery by computers is expected in the near future.

## 1. Introduction

Artificial intelligence (AI) plays an important role in daily life. Significant achievements in numerous different areas such as image and speech recognition, natural language processing etc. have emerged [1,2,3]. Some of the progress in the field is highlighted by computers beating world class players in chess and in Go. While Deep Blue, beating world chess champion Kasparov in 1997, used a set of hard-coded rules and brute force computing power, Alpha Go has learned from playing against itself and won against the world strongest Go player [4,5].

Artificial intelligence is considered as intelligence demonstrated by machines. This term is used, when a machine shows cognitive behavior associated with humans, such as learning or problem solving [6]. AI comprises technologies like machine learning, which are well established for learning and prediction of novel properties. In particular, artificial neural networks, such as deep neural networks (DNN) or recurrent neural networks (RNN) drive the evolution of artificial intelligence.

In pharmaceutical research, novel artificial intelligence technologies received wide interest, when deep learning architectures demonstrated superior results in property prediction. In the Merck Kaggle [7] and the NIH Tox21 challenge [8], deep neural networks showed improved predictivity in comparison to baseline machine learning methods. In the meantime, the scope of AI applications for early drug discovery has been widely increased, for example to de novo design of chemical compounds and peptides as well as to synthesis planning.

Recently, numerous reviews have been published comprising good introductions into the field [9,10,11,12,13,14,15,16,17,18]. Here, we want to focus on recent developments of artificial intelligence in the field of property or activity prediction, de novo design and retrosynthetic approaches.

## 2. Artificial Intelligence in Property Prediction

In drug discovery, clinical candidate molecules must meet a set of different criteria. Next to the right potency for the biological target, the compound should be rather selective against undesired targets and also exhibit good physicochemical as well as ADMET properties (absorption, distribution, metabolism, excretion and toxicity properties). Therefore, compound optimization is a multidimensional challenge. Numerous in-silico prediction methods are applied along the optimization process for efficient compound design. In particular, several machine learning technologies have been successfully used, such as support vector machines (SVM) [19], Random Forests (RF) [20,21] or Bayesian learning [22,23]. 

One important aspect of the success of machine learning for property prediction is access to large datasets, which is a prerequisite for applying AI. In pharmaceutical industry, large datasets are collected during compound optimization for many different properties. Such large datasets for targets and antitargets are available across different chemical series and are systematically used for training machine learning models to drive compound optimization. 

Prediction of activities against different kinases is an illustrative example. Selectivity profiling in different kinase projects generates larger datasets, which have been systematically used for model generation. For Profiling-QSAR [24], binary Bayesian QSAR models were generated from a large, but sparsely populated data matrix of 130,000 compounds on 92 different kinases. These models are applied to novel compounds to generate an affinity fingerprint, which is used to train models for prediction of biological activity against new kinases with relatively few data points. Models are iteratively refined with new experimental data. Thus, machine learning has become part of an iterative approach to discover novel kinase inhibitors. 

In another example of predicting kinase activities Random Forest models could be successfully derived for ~200 different kinases combining publically available datasets with in-house datasets [25]. Random Forest models showed a better performance than other machine learning technologies. Only a DNN showed comparable performance with better sensitivity but worse specificity. Nevertheless, the authors preferred the Random Forest models since they are easier to train. Several recent reviews summarize numerous different additional aspects of machine learning [26,27,28,29]. 

In the public domain large datasets are available and can be used to derive machine learning models for the prediction of cross target activities [30,31,32,33,34]. These models can be applied to drug repurposing, the identification of new targets for an existing drug. Successful applications for repurposing of compounds have been shown using the SEA (Similarity Ensemble Approach) methodology [35]. SEA is a similarity based method, in which ensembles of ligands for each target are compared with each other. Similarities are compared to a distribution obtained from random comparisons to judge the significance of the observed similarities against a random distribution. For repurposing of a ligand, the analysis can also be done with a single molecule queried against an ensemble of ligands for each protein target. 

**Figure 1:**
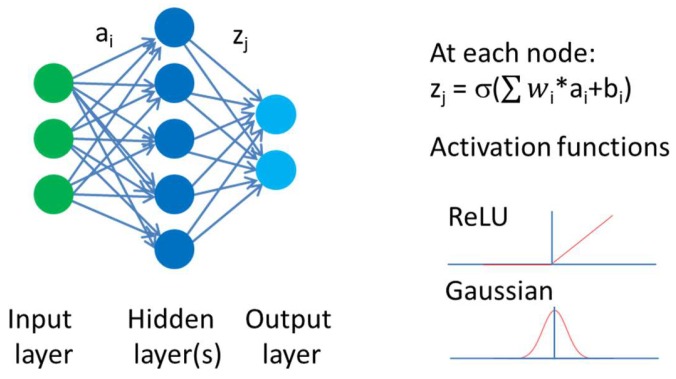
Neurons are connected to each other. Incoming signals are multiplied by a weight. The output signal z_j_ is given by the sum of this products plus a bias transformed by an activation function. Examples of activation functions are graphically shown, like the rectified linear unit (ReLU) or the Gaussian function. For each neuron in the neural net, weights and biases need to be trained. Deep neural networks have several hidden layers with many neurons. The number of neurons typically varies between different layers.

Stimulated by the success of the Kaggle competition, deep neural networks have been used in numerous property prediction problems. Deep neural networks belong to the class of artificial neural networks, which are brain-inspired systems. Multiple nodes, also called neurons, are interconnected like the neurons in the brain. Signals coming in from different nodes are transformed and cascaded to the neurons of the next layer as illustrated in Figure 1. Layers between the input and output layer are called hidden layers. During training of a neural network, weights and biases at the different nodes are adjusted. Deep neural networks are using a significantly larger number of hidden layers and nodes than shallow architectures. Thus, a large number of parameters have to be fitted during the training of the neural network. Therefore, increases in compute power as well as a number of algorithmic improvements were necessary to address the overfitting problem such as dropout [36] or use of rectified linear units to address the vanishing gradient problem [37].

DNNs have been used in numerous examples for property prediction. In many of these studies a comparison to other machine learning approaches has been performed indicating, that DNNs show comparable or better performance than other machine learning approaches, e.g., for different properties ranging from biological activity prediction, ADMET properties to physicochemical parameters. For example, in the Kaggle competition, the DNN shows a better performance for 13 of the 15 assays than a Random Forest approach using 2D topological descriptors [7]. The study revealed that the performance of the DNN is variable, depending on the hyperparameters used, such as the architecture of the network (number of hidden layers as well as the number of neurons in each layer) and the activation function. Definition of a reasonable parameter set is crucial to achieve good performance. 

In another study, a broad dataset from ChEMBL [38] was used comprising more than 5000 different assays and almost 750,000 compounds using Extend Connectivity Fingerprint (ECFP4) [39]. Again, DNNs outperformed several other machine learning methods used for comparison with respect to the area under the ROC curve.

Lenselik et al. performed a large benchmark study on a dataset from ChEMBL coming to a similar conclusion of better performance of the DNN methodology [40]. In this study temporal validation was used for performance comparison where training and test data are split according to publication date. This way of performance measurement is more stringent [41]. In temporal validation performance measures are significantly smaller than in the random split approach, which is probably closer to real life predictivity.

Korotcov et al. compared DNNs to other machine learning algorithms for diverse endpoints comprising biological activity, solubility and ADME properties [42]. In this study, Functional Class Fingerprint (FCFP6) fingerprints were used. The DNN performed better than the SVM approach, which in turn was superior to other machine learning technologies tested. Another interesting aspect of that study revealed that the performance and sensitivity rankings depend on the applied metrices.

**Figure 2:**
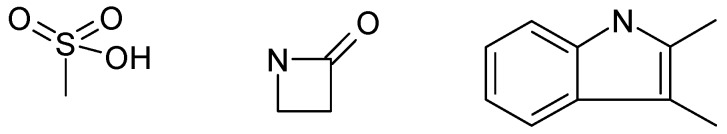
Toxicophoric features identified from the Tox21 dataset by the neural network [8].

Deep learning has also been applied to prediction of toxicity. Results from the Tox21 competition showed, that DNN shows good predictivity on 12 different toxic endpoints [8]. In this study, some emphasis was given to the selection of the molecular descriptors. Absence or presence of known toxicophores was included as one descriptor set in addition to physicochemical descriptors and ECFP type fingerprints. The authors demonstrate, that the DNN is capable of extracting molecular features, which are supposedly associated with known toxicophoric elements, illustrating, that such networks appear to learn more abstract representations in the different hidden layers. Figure 2 gives examples of such features detected by the network. While it is promising, that relevant structural elements can be derived from a DNN, the shown fragments are certainly too generic to be applied to drug discovery without human expertise in the field of toxicology. Additionally, the composition of the training dataset has a strong influence on predictivity and applicability domain of the model as well as the representation learnt by the network, creating a high barrier to such automated learnings. The DeepTox pipeline uses an ensemble of different models, but is dominated by DNN predictions. It outperformed other machine learning approaches in 9 out of 12 toxic endpoints. 

**Figure 3:**
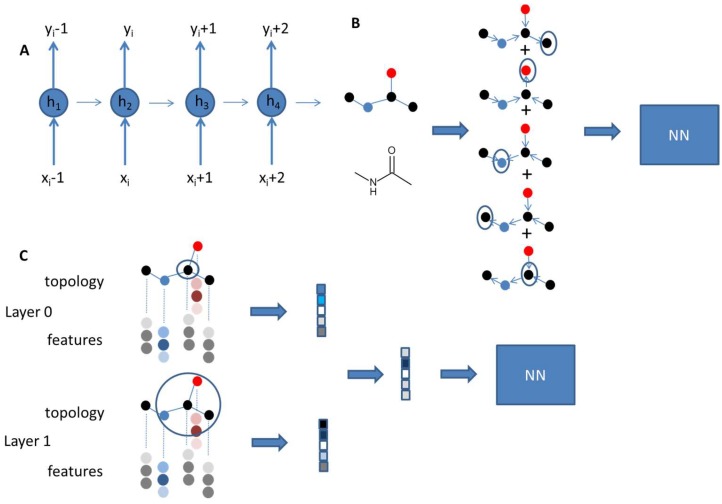
(**A**) Recurrent neural networks (RNNs) use sequential data. The output for the next element depends on the previous element. Thus, RNNs have a memory. h_i_ represent the hidden state at each neuron. They are updated based on the input x and the hidden state from the previous neuron. (**B**) In the UG-RNN approach, molecules are described as undirected graphs and fed into a RNN. Each vertex of a molecular graph is selected as a root node and becomes the endpoint of a directed graph. Output for all nodes is traversed along the graph until the root node is reached. All signals are summed to give the final output of the RNN, which enters into the NN for property training. (**C**) Graph convolutional models use the molecular graph. For each atom a feature vector is defined and used to pass on information for the neighboring atoms. In analogy to circular fingerprints different layers of neighboring atoms are passed through convolutional networks. Summation of the different atomic layers for all atoms results in the final vector entering the neural network for training.

Another example for the prediction of toxic endpoints has been given for the prediction of drug-induced liver injury (DILI) [43]. In this example, the network was trained on 475 compounds and performance was tested on 198 compounds. Good statistical parameters could be achieved for the predicition of drug-induced liver toxicity with accuracy of 86.9%, sensitivity of 82.5%, specificity of 92.9%, and AUC of 0.955. Molecular descriptors from PaDEL [44] and Mold [45] were used as well as a molecular description derived from the UG-RNN method for structural encoding [46] in combination with a line bisection method [47]. In the UG-RNN method, the descriptor is derived from the chemical structures captured as undirected graphs (UGs). Heavy atoms are represented as nodes and bonds as edges. The graph is fed into a recursive neuronal network (RNN) (Figure 3). 

All edges are directed towards the selected root node along the shortest path to cascade vector-encoded atom information to the root node. Every atom becomes the root node. The final output is summed over the different iterations. The UG-RNN derived descriptors show significantly better performance than the other two descriptor sets. 

Using neural networks for encoding of molecular structures is a novel development in the cheminformatics field. While most of the examples described so far, use classical descriptors, more and more implementations allow selection of the chemical descriptor by the neural net. The idea is that the neural network can learn the representation which is best suited for the actual problem in question. Several ways have been described so far. Some of these approaches are shortly described in Wu et al. [48]. Graph convolutional (GC) models are derived from the concept of circular fingerprints [49]. Information is added by adding information from distant atoms in growing out along certain bond distances. These iterations are done for each atom and finally merged into a fixed length vector, which enters a neural network for property prediction. In graph convolutional models the molecular description layer is part of the differentiable network (Figure 3). Thus, training of the neuronal net also optimizes a useful representation of molecules suited to the task at hand. 

In several examples, it was shown, that this training approach indeed improves predictivity for several properties. Duvenaud et al. showed improved performance for a solubility dataset and photovoltaic efficiency, while a biological activity prediction did not benefit from this approach. Additionally, the authors could identify molecular descriptors which are relevant for the different properties [50]. Li et al. introduced a dummy supernode as a new layer for molecular representation and could show good results on datasets from the MoleculeNet [48] dataset [51]. Other versions of convolutional networks have also been introduced. Graph convolution models using a simple molecular graph description show good results already. Kearnes et al. conclude that current graph convolutional models do not consistently outperform classical descriptors, but are a valuable extension of method repertoire, which provide novel flexibility, since the model can pick relevant molecular features and thus give access to a large descriptor space [52]. Related to work on image recognition, molecular structures have also been captured as images and fed into the network. This representation slightly outperformed a network trained on ECFP fingerprints on solubility and activity datasets, but performed slightly worse in toxicity prediction [53].

QSAR and machine learning models are usually trained for one endpoint, although multiple endpoints can be used. DNNs offer the possibility to systematically combine predictions for several endpoints as multitask learning. Multitask learning can improve prediction quality as has been shown by several studies, which compared the performance of singletask vs. multitask models. Ramsundar et al. analysed the benefit of multitask learning for a dataset containing up to 200 assays [54]. Overall, an increase of the performance of the models is observed with multitask learning, while it appears to be stronger for certain tasks. A dataset appears to show improved performance when it shares many active compounds with other tasks. In addition, both, the amount of data and the number of tasks were described to beneficially influence multitask learning. In another study on industry sized ADME datasets beneficial effects for multitask learning could be identified as well, although the improvement appears to be highly dataset dependent [55]. 

Conclusions about the best performance were also observed to be dependent on temporal or random split type validation. Simply adding massive amount of data does not guarantee a positive effect on predictivity. While multitask learning appears to have beneficial effects on a wide variety of different datasets, there are also examples of a drop in predictivity for some endpoints [56]. Xu et al. showed, that in multitask learning some information is “borrowed” from other endpoints, which leads to improved predictions [57]. According to the authors, an improved r^2^ can be observed, when compounds in the training data for one endpoint are similar to compounds from the test data for a second endpoint and activities are correlated (positively or negatively). If activities are uncorrelated, a tendency for a decrease of r^2^ was observed. If molecules between two endpoints are different from each other, no significant effect on r^2^ can be expected from multitask learning.

Bajorath et al. used a set of about 100,000 compounds to develop a model prediction panel against 53 different targets [58]. Overall, good predictivity was achieved. Interestingly, the comparison between DNNs and other machine learning technologies does not yield any superior performance of the deep learning approach. The authors discuss, that the dataset is relatively small and thus might not be suited to demonstrate the full potential of DNNs.

Deep learning has also been used to predict potential energies of small organic molecules replacing a computational demanding quantum chemical calculation by a fast machine learning method. For large datasets, quantum chemically derived DFT potential energies have been calculated and used to train deep neuronal nets. The network was possible to predict the potential energy, called ANI-1, even for test molecules with higher molecular weight than the training set molecules [59].

Deep learning has been extensively validated for a number of different datasets and learning tasks. In a number of comparisons, DNNs show an improvement compared to well established machine learning technologies. This has also been demonstrated in a recent large-scale comparison of different methods, in which the performance of DNNs was described as comparable to in-vitro assays [60]. Nevertheless, many of the studies are performed retrospectively to show the applicability of deep learning architectures for property prediction and to compare the method to established machine learning algorithms. Often, public datasets like ChEMBL are used. In ChEMBL, biological data are often only available for one target resulting in a sparsely populated matrix, making cross-target learnings a significant challenge. Thus, it still remains to be seen, in which scenarios, DNNs clearly outperform other machine learning approaches, in particular since training and parameter optimization is less demanding for many other machine learning methods. A promising development is the self-encoding of the compound description by the learning engine, which will offer problem-dependent optimized compound descriptions.

## 3. Artificial Intelligence for de novo Design

**Figure 4:**
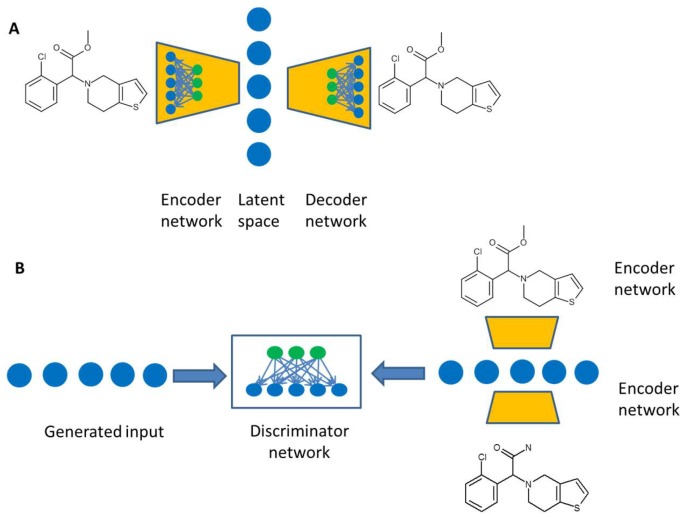
(**A**) A variational autoencoder consists of two neural networks. The encoder network transforms the molecular description into a description vector, the latent space, while the decoder network is trained to translate a latent space vector into a molecule. (**B**) The adversarial autoencoder comprises a standard autoencoder, which learns to generate chemical structures. The discriminator network compares descriptions from a defined distribution to structures generated from the autoencoder.

De novo design aiming to generate new active molecules without reference compounds was developed approximately 25 years ago. Numerous approaches and software solutions have been introduced [61,62]. But de novo design has not seen a widespread use in drug discovery. This is at least partially related to the generation of compounds, which are synthetically difficult to access. The field has seen some revival recently due to developments in the field of artificial intelligence. An interesting approach is the variational autoencoder (Figure 4), which consists of two neural networks, an encoder network and a decoder network [63]. The encoder network translates the chemical structures defined by SMILES representation into a real-value continuous vector as a latent space. The decoder part is capable to translate vectors from that latent space into chemical structures. This feature was used to search for optimal solutions in latent space by an in-silico model and to back translate these vectors into real molecules by the decoder network. For most back translations one molecule dominates, but slight structural modifications exist with smaller probability. The authors used the latent space representation to train a model based on the QED drug-likeness score [64] and the synthetic accessibility score SAS [65]. A path of molecules with improved target properties could be obtained. In another publication, the performance of such a variational autoencoder was compared to an adversarial autoencoder [66]. The adversarial autoencoder consists of a generative model producing novel chemical structures. A second discriminative adversarial model is trained to tell apart real molecules from generated ones, while the generative model tries to fool the discriminative one. The adversarial autoencoder produced significantly more valid structures than the variational autoencoder in generation mode. In combination with an in-silico model novel structures predicted to be active against the dopamine receptor type 2 could be obtained. Kadurin et al. used a generative adversarial network (GAN) to suggest compounds with putative anticancer properties [67]. 

Recursive neural networks (RNN) have also been successfully used for de novo design. Originally, they have been established in the area of natural language processing [68]. RNNs take sequential information as input. Since SMILES strings encode chemical structures in a sequence of letters, RNNs have been used for generation of chemical structures. To teach the neural network the grammar of SMILES strings, RNNs are trained with a large set of chemical compounds taken from existing compound collections such as ChEMBL or commercially available compounds. It was shown, that RNNs are capable of producing a large fraction of valid SMILES strings [69,70]. The same approach was also successfully used for the generation of novel peptide structures [71]. Reinforcement learning was successfully applied to bias the generated compounds towards desired properties [72]. 

Transfer learning was used as another strategy to generate novel chemical structures with a desired biological activity. In the first step, the network is trained to learn the SMILES grammar with a large training set. In the second step, the training is continued with compounds having the desired activity. Few additionally epochs of training are sufficient to bias the generation of novel compounds into a chemical space occupied by active molecules [69]. Based on such an approach five molecules were synthesized and the design activity could be confirmed for four molecules against nuclear hormone receptors [73]. 

Several different architectures have been implemented, which are capable of generating valid, meaningful novel structures. The novel chemical space can be explored by these methods with the property distribution of the generated molecules being similar to the training space. The first prospective application for this methodology was successful with 4 out of 5 molecules showing the desired activity. Nevertheless, more experience need to be gained with respect to the size of the chemical space sampled and chemical feasibility of the proposed molecules.

## 4. Artificial Intelligence for Synthesis Planning

Organic synthesis is a critical part of any small molecule drug discovery program. New molecules are synthesized to progress along the compound optimization path and to identify molecules with improved properties. In certain situations, synthetic challenges restrict the available chemical space accessible for design molecules. Therefore synthesis planning is a key discipline in drug discovery. Accordingly, numerous computational approaches have been developed to assist synthesis planning. Three different aspects can be distinguished: prediction of the outcome of a reaction with a given set of educts, prediction of the yield of a chemical reactions as well as retrosynthetic planning. In particular, retrosynthetic planning is dominated by knowledge-based systems, which are built on expert-derived rules or automatically extracted rules from reaction databases [74,75,76,77]. 

Recently, a number of machine learning based approaches have been described for forward synthesis prediction. Forward synthesis prediction offers the ranking of synthetic routes from retrosynthetic analysis. In one type of approaches, quantum chemical descriptors have been combined with manual encoded rules and machine learning to predict a reaction and its product(s). [78,79,80]. The methodology has recently been extended to predict multi-step reactions [81]. In another approach [82], a deep neural network has been trained with a set of millions of reactions extracted from Reaxys [83]. The described network outperforms an expert system used for comparison. For reactions in the automatically derived rule set of 8720 templates, the authors report 78% accuracy for the best network. 

Public reaction databases do not contain examples of failed chemical reactions, which is a clear limitation for machine learning approaches. Therefore, in another example, the dataset was augmented with chemical plausible negative examples [84]. At first possible reactions are selected and ranked by the neural network. Based on a training set of 15,000 reactions from granted US patents, the major reaction product was correctly identified top ranked in 71.8% in a 5-fold cross-validation experiment. In a subsequent publication, the authors use a template-free approach to improve coverage of chemical reactions [85]. Forward prediction of chemical reactions based on machine learning shows good performance in published validation studies. Nevertheless, some aspects need further consideration in future developments, such as the inclusion of reaction conditions, used catalysts etc.

Artificial intelligence has also been described for retrosynthetic analysis. Liu et al. used a sequence-to-sequence based model for retrosynthetic reaction prediction. Reactants and products are coded by SMILES strings for RNNs and coupled to each other in an encoder-decoder architecture. The training set spans 10 broad reaction types such as C-C bond formation, reductions, oxidations, heteroatom alkylation etc. and comprises 50,000 reactions from US patent literature [86]. The performance of the technology overall was comparable to rule-based expert systems, but large differences have been observed over different reaction classes. In a different approach recommender systems have been used to identify reactants yielding a desired product in combination with a chemical reaction graph [87]. Nevertheless, AUCs obtained in the validation indicated, that further improvement needs to be done. 

The combination of three deep neural networks with a Monte Carlo tree search for retrosynthetic prediction yielded an excellent performance [88]. Training and test dataset were extracted from the entire Reaxys database and were split in time. For a test set of 497 diverse molecules, synthesized after 2015, over 80% correct synthetic routes were proposed. According to a blind test, medicinal chemists prefer the route proposed by this methodology over proposals from rule-based approaches.

Machine learning-based approaches can mine large datasets humans cannot handle in an unbiased manner. For synthesis planning, the combination of knowledge-based and machine learning approaches for prediction of chemical reactions turned out to be quite powerful. On the other hand, the purely machine-based approach capitalizing on a large reaction database shows excellent performance. Nevertheless, one limitation remains for in-silico tools, the capability to propose and develop novel chemical reactions. Here, a detailed analysis is necessary and will rely on the use of quantum chemical methods in the future [89].

## 5. Conclusions and Outlook

Artificial intelligence has received much attention recently and also has entered the field of drug discovery successfully. Many machine learning methods, such as QSAR methods, SVMs or Random Forests are well-established in the drug discovery process. Novel algorithms based on neural networks, such as deep neural networks, offer further improvements for property predictions, as has been shown in numerous benchmark studies comparing deep learning to classical machine learning. The applicability of these novel algorithms for a number of different applications has been demonstrated including physicochemical properties as well as biological activities, toxicity etc. Some benefit from multitask learning has also been shown, where the prediction of related properties appears to benefit from joint learning. Future improvement can be expected from the capability of learning a chemical representation which is adapted to the problem at hand. First efforts have been taken, to identify relevant chemical features from such representations, which also points to one major challenge of these algorithms, which is their “black box” character. It is very difficult to extract from deep neural networks, why certain compounds are predicted to be good. This becomes relevant, if synthesis resources are more and more guided by artificial intelligence.

On the other hand, the effort of training such models will be increasing compared to established machine learning technologies. A large number of hyperparameters need to be tuned and optimized for good performance, although some studies indicate, that some reasonable parameter set can be used for starting the optimization. 

The application of artificial intelligence for drug discovery benefits strongly from open source implementations, which provide access to software libraries allowing implementation of complex neural networks. Accordingly, open source libraries like Tensorflow [90] or Keras [91] are frequently used to implement different neural network architectures in drug discovery. Additionally, the Deepchem library provides a wrapper around Tensorflow that simplifies processing of chemical structures [92].

The scope of applications of artificial intelligence systems has been largely increased over recent years, now also comprising de novo design or retrosynthetic analysis, highlighting, that we will see more and more applications in areas where large datasets are available. With progress in these different areas, we can expect a tendency towards more and more automated drug discovery done by computers. In particular, large progress in robotics will accelerate this development. Nevertheless, artificial intelligence is far from being perfect. Other technologies with sound theoretical background will remain important, in particular, since they also benefit from increase in compute power, thus larger systems can be simulated with more accurate methods. Furthermore, there are still missing areas, novel ideas, which cannot be learned from data, giving a combination of human and machine intelligence a good perspective.
